# Detection of human intestinal protozoan parasites in vegetables and fruits: a review

**DOI:** 10.1186/s13071-020-04255-3

**Published:** 2020-07-29

**Authors:** Junqiang Li, Zhenzhen Wang, Md Robiul Karim, Longxian Zhang

**Affiliations:** 1 Academy of Chinese Medical Sciences, Henan University of Chinese Medicine, Zhengzhou, 450046 China; 2 College of Animal Science and Veterinary Medicine, Henan Agricultural University, Zhengzhou, 450046 China; 3 Department of Medicine, Bangabandhu Sheikh Mujibur Rahman Agricultural University, Gazipur, 1706 Bangladesh

**Keywords:** Intestinal protozoans, Detection methods, Vegetables, Fruits, Contamination

## Abstract

Diarrheal diseases caused by intestinal protozoan parasites are a major food-borne public health problem across the world. Vegetables and fruits provide important nutrients and minerals, but are also common sources of some food-borne human pathogenic microorganisms. The contamination of raw vegetables and fruits with human pathogenic parasites are now a global public health threat, despite the health benefits of these foods in non-pharmacological prophylaxes against diseases. A large number of reports have documented the contamination of vegetables or fruits with human pathogenic microorganisms. In this paper, we reviewed the contamination and detection methods of human pathogenic intestinal protozoans that are frequently recovered from raw vegetables and fruits. The protozoan parasites include *Cryptosporidium* spp., *Giardia duodenalis*, *Cyclospora cayetanensis*, *Entamoeba* spp., *Toxoplasma gondii*, *Balantioides coli*, *Blastocystis* sp., *Cystoisospora belli* and *Enterocytozoon bieneusi*. The risk factors involved in the contamination of vegetables and fruits with parasites are also assessed. 
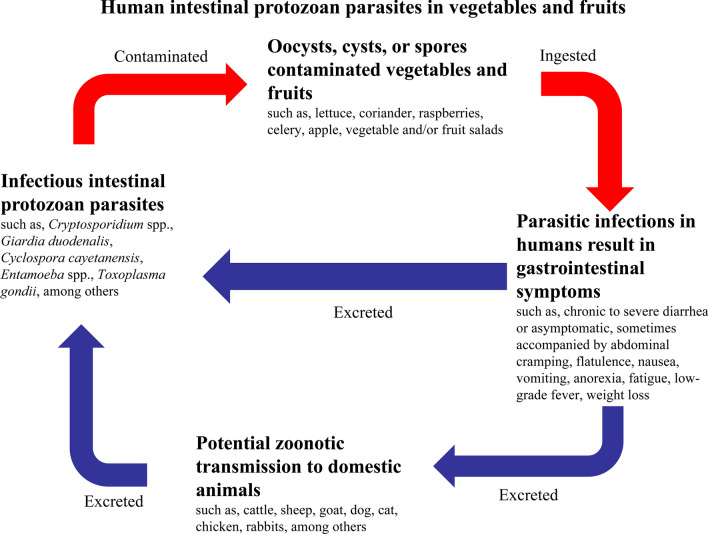

## Background

Nearly 1.7 billion cases of diarrheal disease are reported globally every year, imposing an annual socioeconomic burden on health services of 72.8 million disability-adjusted life years [1, 2]. A number of pathogens are responsible for causing diarrheal diseases, among which intestinal protozoan parasites are important contributors that can be transmitted by ingestion of the contaminated food [3, 4]. The intestinal protozoan infections are characterized by chronic to severe diarrhea, sometimes accompanied by abdominal cramping, flatulence, nausea, vomiting, anorexia, fatigue, low-grade fever and weight loss [5–7].

Vegetables and fruits provide important nutrients to humans, including various essential vitamins and minerals [8]. The ingestion of raw vegetables and fruits appear to be a quick, easy, and healthy source of nutrition. However, these fresh vegetables and fruits can be an important source of some food-borne pathogenic microorganisms, if they are contaminated [9, 10]. The contamination of raw vegetables and fruits with human parasites has recently been recognized as a global threat, despite the health benefits of these foods in non-pharmacological prophylaxes against diseases.

A number of studies documented the contamination of vegetables and fruits with human pathogenic microorganisms [11–15]. In this paper, we reviewed the detection methods and contamination of some human pathogenic intestinal protozoans that are frequently recovered from raw vegetables and fruits. The protozoan parasites include *Cryptosporidium* spp., *Giardia duodenalis*, *Cyclospora cayetanensis*, *Entamoeba* spp., *Toxoplasma gondii*, *Balantioides coli*, *Blastocystis* sp., *Cystoisospora belli* and *Enterocytozoon bieneusi*.

We searched PubMed and Web of Science databases, with no language restrictions, using the following search terms: ‘*Cryptosporidium*’ or ‘*Giardia*’ or ‘*Cyclospora*’ or ‘*Entamoeba*’ or ‘*Toxoplasma gondii*’ or ‘*Balantioides coli*’ or ‘*Blastocystis* sp.’ or ‘*Cystoisospora belli*’ or ‘*Isospora belli*’ or ‘microsporidian’ and ‘vegetable’ or ‘fruit’. Articles were screened using Endnote X9. For articles whose full text was unavailable or that were published in other languages, the titles and abstracts in English were screened. Articles published up to December 31st 2019 were included in this review.

### Detection methods of intestinal protozoan parasites contaminating vegetables and fruits

The recovery of parasitic eggs/oocysts/cysts from contaminated vegetables and fruits with proper methods is the first and an important way for the detection of contaminating intestinal protozoa. The methods or techniques for the detection of *Cryptosporidium* in food samples were well reviewed by Ahmed and Karanis in 2018 [16].

Generally, a washing procedure is the first step in any recovery process. Several elution strategies have been used to isolate the parasites from vegetables and fruits. A portion (usually 50–250 g) of each vegetable or fruit sample is washed separately in a container containing some chemical solutions. The most widely used solutions are normal saline [14, 17–20] and phosphate-buffered saline [12, 21–24]. The commonly used solutions are glycine [11, 25], sodium dodecyl sulfate [26], Alconox^®^ [27], and Tween 80 [28]. Other unusual solutions, such as 10% formal saline [29] and 0.1% peptone water [30] are also reported to isolate the contaminating parasites. Different elution methods can lead to variable recovery rates for parasites from contaminated vegetables or fruits, however, the Alconox^®^ solution was reported to be more effective than the other commonly used solutions [27, 31].

The isolation of the detergent solution sediments is the second key step in parasite detection. Two methods are commonly used to obtain these concentrated sediments. One is the overnight sedimentation of the washing solution [19, 30]. The supernatant is discarded and the sediment is then transferred to a new tube to remove any unwanted material [32]. The other is membrane filtration (more commonly and effectively used), in which the deposit is collected by centrifugation. Membrane filtration devices include stomacher bags [23, 30], zipper bags [22, 24], sieves [18], gauze [21], or cellulose acetate membranes [28].

Finally, the sediment or deposit is screened with light microscopy, staining, immunofluorescence microscopy, or PCR to detect any parasite. More than one smear slide is usually prepared for each specimen to allow its precise detection [12, 26]. Oocysts or cysts can be detected microscopically based on their morphological features [14, 17, 20, 29], using Lugol’s iodine [12, 14, 29] or modified Ziehl-Neelsen staining (or any other staining technique) [14, 19, 26]. The extraction of the parasitic DNA from the sediment, followed by the PCR amplification of specific genes, is also efficiently used for the protozoan detection in vegetable and fruit samples [22, 24].

### Contamination of vegetables and fruits with intestinal protozoan parasites

#### *Cryptosporidium* contamination

*Cryptosporidium* spp. are widespread protozoan parasites that infect humans and animals, and the second commonest cause of diarrhea in children after rotavirus [9]. *Cryptosporidium* is characterized by its extensive genetic variation that results in the existence of 38 species and more than 60 genotypes of this parasite [33]. At least 20 distinct species cause moderate or severe infections in humans, of which *C. hominis* and *C. parvum* are the major causative agents [34].

The detection of *Cryptosporidium* oocysts in vegetable and fruit samples with light microscopy is simple, convenient, and direct [13, 16], but it requires a high level of expertise to interpret the slides, while an immunofluorescence assay is standard practice and more sensitive [16]. Immunomagnetic separation (IMS) is used to concentrate *Cryptosporidium* oocysts for the efficient detection by microscopy or PCR [12, 25, 35]. The PCR amplification and sequencing of specific genes of *Cryptosporidium* recovered from contaminated vegetables and fruits is the most precise method of identification of human pathogenic and zoonotic species (e.g., [13, 23–25]. However, PCR is commonly used in developed countries, but most surveillance studies in developing countries involve microscopy.

The contamination of vegetables and fruits with *Cryptosporidium* spp. has been documented in many countries (Table 1), and the average prevalence is calculated as 6.0% (375/6210; 95% confidence interval, CI: 5.4–6.6%). Among the *Cryptosporidium* species, *C. parvum*, *C. hominis*, and *C. ubiquitum* were detected in the contaminated vegetable and fruit samples [12, 23, 25, 36]. The *Cryptosporidium* species are important human pathogens and major causes of human cryptosporidiosis, representing a threat to public health through food as a vehicle.

**Table 1:** Contamination of vegetables and fruits by *Cryptosporidium* spp.

Location	Detection method	Vegetable or fruit item	No. of samples tested	No. of positive samples (%)	*Cryptosporidium* species (*n*)	References
Brazil	PCR	Vegetables	21	2 (9.5)	*Cryptosporidium* spp. (1); *C. parvum* (1)	[45]
China	PCR	Lettuce	200	0		[36]
		Coriander	152	0		
		Celery	70	0		
		Baby bok choy	59	0		
		Chinese cabbage	47	0		
		Leaf lettuce	44	0		
		Water spinach	28	0		
		Crown daisy	27	0		
		Fennel plant	26	0		
		Endive	25	0		
		Spinach	20	0		
		Schizonepeta	20	0		
		Cabbage	18	0		
		Leaf mustard	11	0		
		Chinese chive	132	1 (0.8)	*C. parvum* (1)	
		Chive	128	0		
		Cucumber	41	0		
		Watermelon	15	0		
		Potato	3	0		
		Bean (kidney/French bean)	28	0		
		Green chili	5	0		
Costa Rica	Direct smear, followed by light microscopy	Cilantro leaves	80	4 (5.0)	*Cryptosporidium* spp. (4)	[79]
		Cilantro roots	80	7 (8.7)	*Cryptosporidium* spp. (7)	
		Lettuce	80	2 (2.5)	*Cryptosporidium* spp. (2)	
		Radish, tomato, cucumbers, carrots	80	1 (1.2)	*Cryptosporidium* spp. (1)	
Costa Rica	Zielh-Nielsen stain, Weber stain	Lettuce	50	7 (14.0)	*Cryptosporidium* spp. (7)	[71]
		Parsley	50	1 (2.0)	*Cryptosporidium* spp. (1)	
		Cilantro	50	1 (2.0)	*Cryptosporidium* spp. (1)	
		Strawberries	50	0		
		Blackberries	50	3 (6.0)	*Cryptosporidium* spp. (3)	
Egypt	Wet mount, Weber modified trichrome, modified Ziehl-Neelsen stains	Fresh fruit juices		61.3	*Cryptosporidium* spp.	[80]
Ethiopia	Modifed Zeihl-Neelsen stain	Fruits and vegetables	360	46 (12.8)	*Cryptosporidium* spp. (46)	[19]
Ethiopia	Modified Ziehl-Neelsen stain	Fruits and vegetables	360	17 (4.7)	*Cryptosporidium* spp. (17)	[32]
Ethiopia	Modified Zeihl-Neelsen stain	Tomato	100	9 (9.0)	*Cryptosporidium* spp. (9)	[14]
		Cabbage	96	0		
		Green pepper	66	2 (3.0)	*Cryptosporidium* spp. (2)	
		Carrot	62	7 (11.3)	*Cryptosporidium* spp. (7)	
		Salad	23	2 (8.7)	*Cryptosporidium* spp. (2)	
Ghana	Ziehl-Neelsen stain	Cabbage	90	18 (20.0)	*Cryptosporidium parvum* (18)	[12]
		Green pepper	55	12 (21.8)	*Cryptosporidium parvum* (12)	
		Carrot	47	6 (12.8)	*Cryptosporidium parvum* (6)	
		Onion	70	9 (12.9)	*Cryptosporidium parvum* (9)	
		Tomato	31	4 (12.9)	*Cryptosporidium parvum* (4)	
		Lettuce	102	18 (17.6)	*Cryptosporidium parvum* (18)	
Ghana	Sediment smears and fluorescence stain	Cabbage	72	12 (16.7)	*Cryptosporidium* spp. (12)	[67]
		Lettuce	72	15 (20.8)	*Cryptosporidium* spp. (15)	
		Carrot	72	4 (5.6)	*Cryptosporidium* spp. (4)	
		Spring onion	72	8 (11.1)	*Cryptosporidium* spp. (8)	
		Tomatoes	72	1 (1.4)	*Cryptosporidium* spp. (1)	
Ghana	Direct wet mount, Trichrome, modified Zielh-Nielsen stain	Tiger nuts	40	12 (30.0)	*Cryptosporidium parvum* (12)	[81]
India	DAPI-stain followed by fluorescence microscopy, and PCR	Cabbage	47	3 (6.4)	*Cryptosporidium parvum* (3)	[13]
		Chili	42	2 (4.8)	*Cryptosporidium* spp. (2)	
		Coriander	28	2 (7.1)	*Cryptosporidium* spp. (2)	
		Cucumber	52	3 (5.8)	*Cryptosporidium parvum* (3)	
		Radish	14	1 (7.1)	*Cryptosporidium* spp. (1)	
		Tomatoes	56	6 (10.7)	*Cryptosporidium* spp. (6)	
Iran	Modified Ziehl-Neelsen acid-fast stain	Mint	82	7 (8.5)	*Cryptosporidium* spp. (7)	[26]
		Leek	90	3 (3.3)	*Cryptosporidium* spp. (3)	
		Cress	90	8 (8.9)	*Cryptosporidium* spp. (8)	
		Green onion	54	8 (14.8)	*Cryptosporidium* spp. (8)	
		Coriander	90	6 (6.7)	*Cryptosporidium* spp. (6)	
		Basil	90	1 (1.1)	*Cryptosporidium* spp. (1)	
Iran	Modified Ziehl-Neelsen satin	Vegetables	34	3 (8.8)	*Cryptosporidium* spp. (3)	[72]
Italy	modified Ziehl-Neelsen stain and PCR	Ready-to-eat packaged salads	648	6 (0.9)	*Cryptosporidium parvum*/*C. ubiquitum* (6)	[23]
Korea	qPCR	Carrots	3	1 (33.3)	*Cryptosporidium parvum* (1)	[22]
		Cabbages	3	1 (33.3)	*Cryptosporidium parvum* (1)	
		Blue berries	3	1 (33.3)	*Cryptosporidium parvum* (1)	
Korea	Multiplex qPCR	Perilla leaves	72	5 (6.9)	*Cryptosporidium* spp. (5)	[24]
		Winter-grown cabbage	70	4 (5.7)	*Cryptosporidium* spp. (4)	
		Chives	73	13 (17.8)	*Cryptosporidium* spp. (13)	
		Sprouts	72	1 (1.4)	*Cryptosporidium* spp. (1)	
		Blueberries	44	3 (6.8)	*Cryptosporidium* spp. (3)	
		Cherry tomatoes	73	5 (6.8)	*Cryptosporidium* spp. (5)	
Norway	Concentrated by IMS, and screening by light microscopy	Alfalfa sprouts	16	0		[35]
		Dill	7	0		
		Lettuce	125	5 (4.0)	*Cryptosporidium* spp. (5)	
		Mung bean sprouts	149	14 (9.4)	*Cryptosporidium* spp. (14)	
		Mushrooms	55	0		
		Parsley	7	0		
		Precut salad mix	38	0		
		Radish sprouts	6	0		
		Raspberries	10	0		
		Strawberries	62	0		
Norway	Concentrated by IMS, and screening by light microscopy	Alfalfa	16	0		[82]
		Mung bean	149	14 (9.4)	*Cryptosporidium* spp. (14)	
		Radish	6	0		
Peru	Direct microscopic observation, acid-fast staining, and immunofluorescent assays	Vegetables		14.5	*Cryptosporidium parvum*	[83]
Poland	Separated by IMS and identified by immunofluorescence and DIC microscopy, and PCR identified	Fresh vegetables	128	6 (4.7)	*Cryptosporidium parvum* or *C. hominis* (6)	[25]
		Fruits	35	0		
Spain	Concentrated by IMS and stain oocysts for immunofluorescence assay	Chinese cabbage	6	2 (33.3)	*Cryptosporidium* spp. (2)	[11]
		Lollo rosso lettuce	4	3 (75.0)	*Cryptosporidium* spp. (3)	
		Romaine lettuce	9	7 (77.8)	*Cryptosporidium* spp. (7)	
Total			6210	375 (6.0)		

### *Giardia duodenalis* contamination

*Giardia duodenalis* (synonyms: *G. intestinalis*, *G. lamblia*) is a non-invasive protozoan parasite that adhere to and colonize the upper small intestine, causing acute watery diarrhea in humans and animals [37]. It is an important zoonotic protozoan and the main cause of human giardiasis, which therefore represents a threat to public health [38]. Eight genetically distinct assemblages (A to H) of *G. duodenalis* have been defined, with the occurrence of zoonotic assemblages A and B in both humans and animals. However, the other assemblages are mostly specific to animal hosts [38]. This parasite is estimated to cause ~28.2 million cases of diarrhea annually through the ingestion of contaminated foods [7]. The outbreaks of giardiasis have also been associated with a variety of processed foods. Human infections of *G. duodenalis* are often associated with the consumption of contaminated raw vegetables and fruits [39–41].

*Giardia duodenalis* cysts can be detected with light microscopy based on their morphological features [19, 42, 43], and staining with typical Lugol’s iodine is universally used for the detection of *G. duodenalis* cysts [12, 14, 17, 18, 29]. However, an immunofluorescence assay is usually applied for the detection of *Giardia* cysts in food items with more sensitivity [7]. The IMS method is also applied to concentrate *G. duodenalis* cysts for further detection [11, 35]. The PCR amplification and sequencing of specific *G. duodenalis* genes recovered from contaminated food are also commonly used for the confirmatory detection of this parasite (e.g. [28, 39, 44]).

The contamination of vegetables and fruits with *G. duodenalis* cysts has been reported in many countries (Table 2), and the average prevalence is estimated as 4.8% (276/5739; 95% CI: 4.2–5.4%). In contaminated vegetable and fruit samples, *G. duodenalis* zoonotic assemblages A and B were commonly detected [23, 28, 39, 44, 45].

**Table 2:** Contamination of vegetables and fruits with *Giardia duodenalis*

Location	Detection method	Vegetable or fruit item	No. of samples tested	No. of positive samples (%)	*Giardia duodenalis* assemblages identified (*n*)	References
Bangladesh	Iodine and normal saline wet mount	Vegetables	200	2 (1.0)		[52]
Brazil	PCR	Lettuce and chicory	11	2 (18.2)	Assemblage BIV (2)	[39]
Brazil	Immunofluorescence, PCR	Arugula	4	2 (50.0)	Assemblage AII (2)	[28]
		Chives	12	1 (8.3)	Assemblage AII (1)	
		Crisp lettuce	32	4 (12.5)	Assemblage AII (4)	
		Greens collard	24	1 (4.2)	Assemblage AI (1)	
		Parsley	12	2 (16.7)	Assemblage AII (2)	
		Watercress	12	4 (33.3)	Assemblage AII (4)	
		Wild chicory	12	2 (16.7)	Assemblage AII (2)	
Brazil	Semi-nested PCR	Regular lettuce	60	8 (13.3)	Assemblage AI (4); Assemblage B (1); Assemblage E (1); N/D (2)	[44]
		Crisp lettuce	100	5 (5.0)	Assemblage AI (2); N/D (3)	
		Chicory	60	5 (8.3)	Assemblage AI (3); N/D (2)	
		Rocket	20	1 (5.0)	N/D (1)	
		Kale	20	0		
Brazil	PCR	Vegetables	21	10 (47.6)	Assemblage E (2); N/D (8)	[45]
Brazil	Sediment being stained in Lugolʼs solution	Lettuce	100	0		[15]
		Coriander	100	1 (1.0)		
Costa Rica	Direct smear, followed by light microscopy	Cilantro leaves	80	4 (5.0)		[79]
		Cilantro roots	80	2 (2.5)		
Egypt	Lugol’s iodine stain	Lettuce	101	16 (15.8)		[18]
		Watercress	116	13 (11.2)		
		Parsley	102	12 (11.8)		
		Green onion	103	4 (3.9)		
		Leek	108	2 (1.9)		
Ethiopia	Lugol’s iodine stain	Fruits and vegetables	360	27 (7.5)		[19]
Ethiopia	Sediment smear under light microscope	Fruits and vegetables	360	36 (10.0)		[32]
Ethiopia	Sediment smear under light microscope	Tomatoes	45	1 (2.2)		[43]
		Lettuce	45	4 (8.8)		
		Carrot	45	7 (15.6)		
		Cabbage	45	8 (17.8)		
		Green pepper	45	6 (13.3)		
		Avocado	45	0		
Ethiopia	Sediment smear and Lugol’s iodine stain	Tomato	100	0		[14]
		Cabbage	96	16 (16.7)		
		Green pepper	66	4 (6.1)		
		Carrot	62	4 (6.5)		
		Salad	23	0		
Ghana	Lugol’s iodine stain	Cabbage	90	5 (5.6)		[12]
		Green pepper	55	3 (5.5)		
		Carrot	47	4 (8.5)		
		Onion	70	3 (4.3)		
		Tomato	31	2 (6.5)		
		Lettuce	102	5 (4.9)		
India	DAPI-stain followed by fluorescence microscopy, and PCR	Cabbage	47	1 (2.1)		[13]
		Carrot	25	1 (4.0)		
		Chili	42	4 (9.5)		
		Coriander	28	3 (10.7)		
		Cucumber	52	1 (1.9)	Assemblage D (1)	
		Tomatoes	56	2 (3.6)	Assemblage A (2)	
		Turnip	3	1 (33.3)		
Iran	Lugol’s iodine stain	Vegetables	141	11 (7.8)		[84]
Iran	Sediment smear under light microscopy	Leek	30	3 (10.0)		[42]
		Spring onion	22	0		
		Basil	15	1 (6.7)		
		Parsley	21	0		
		Lettuce	23	0		
		Cress	17	0		
		Spearmint	18	0		
		Tarragon	19	0		
		Coriander	24	0		
		Radish	29	0		
Italy	Lugolʼs iodine satin and PCR	Ready-to-eat packaged salad	648	4 (0.6)	Assemblage A (4)	[23]
Jordan	Lugol’s iodine stain	Lettuce	30	7 (23.3)		[20]
		Tomato	33	2 (6.1)		
		Parsley	42	0		
		Cucumber	28	0		
Norway	Concentrated by IMS, and screening by light microscopy	Alfalfa sprouts	16	0		[35]
		Dill	7	2 (28.6)		
		Lettuce	125	2 (1.6)		
		Mung bean sprouts	149	3 (2.0)		
		Mushrooms	55	0		
		Parsley	7	0		
		Precut salad mix	38	0		
		Radish sprouts	6	1 (16.7)		
		Raspberries	10	0		
		Strawberries	62	2 (3.2)		
Norway	Concentrated by IMS, and screening by light microscopy	Alfalfa	16	0		[82]
		Mung bean	149	3 (2.0)		
		Radish	6	1 (16.7)		
Saudi Arabia	Lugol’s iodine stain	Green onion	50	0		[17]
		Watercress	50	0		
		Lettuce	50	0		
		Cucumber	50	0		
		Cabbage	50	0		
		Pea	50	0		
		Tomato	50	0		
		Carrot	50	4 (8.0)		
Spain	Concentrated by IMS and stain cysts for immunofluorescence assay	Chinese cabbage	6	2 (33.3)		[11]
		Lollo rosso lettuce	4	3 (75.0)		
		Romaine lettuce	9	5 (55.6)		
Sudan	Lugol’s iodine stain	Tomatoes	36	1 (2.8)		[29]
		Cucumber	12	0		
		Armenian cucumber	16	0		
		Green pepper	25	1 (4.0)		
		Cayenne pepper	7	0		
		Radish	24	1 (4.2)		
		Beet	19	0		
		Watercress	23	2 (8.7)		
		Lettuce	11	1 (9.1)		
		Green onion	36	1 (2.8)		
		Carrot	50	1 (2.0)		
Total			5739	276 (4.8)		

*Giardia duodenalis, G. intestinalis, G. lamblia*

### *Cyclospora cayetanensis* contamination

*Cyclospora cayetanensis* is another important protist parasite, usually transmitted *via* food that causes human gastrointestinal cyclosporiasis [5, 46]. Globally, *C. cayentanesis* is an important food-borne human protozoan [5, 46]. Many reports have documented the food-borne cyclosporiasis outbreaks that were associated with the consumption of contaminated raw vegetables or fruits.

*Cyclospora cayetanensis* oocysts can be detected simply and directly with light microscopy provided that there are a large number of oocysts present in the vegetables and fruits [23, 37]. Modified Ziehl-Neelsen staining, and autofluorescence or immunofluorescence assays are also commonly used for their detection [12, 14, 19, 47]; however, there are no immunofluorescence assays commercially available for *Cyclospora*. Furthermore, PCR amplification and sequencing of *C. cayetanensis* genes have currently been used for the specific detection of this organism in contaminated food samples [23, 24, 48].

The contamination of vegetables and fruits with *C. cayetanensis* oocysts have been documented in many countries (Table 3). The average prevalence of *C. cayetanensis* contamination is counted as 3.9% (180/4628; 95% CI: 3.3–4.5%).

**Table 3:** Contamination of vegetables and fruits with *Cyclospora cayetanensis*

Location	Detection method	Vegetable or fruit item	No. of samples tested	No. of positive samples (%)	References
Cameroon	Sediment smear, followed by light microscopy	Green cabbage	30	0	[66]
		Red cabbage	30	0	
		Lettuce	30	10 (33.3)	
		Cucumber	30	0	
		Carrots	30	0	
		Green pepper	30	20 (66.7)	
China	PCR	Lettuce	200	1	[36]
		Coriander	152	0	
		Celery	70	0	
		Baby bok choy	59	0	
		Chinese cabbage	47	0	
		Leaf lettuce	44	1 (2.3)	
		Water spinach	28	0	
		Crown daisy	27	0	
		Fennel plant	26	0	
		Endive	25	0	
		Spinach	20	0	
		Schizonepeta	20	0	
		Cabbage	18	0	
		Leaf mustard	11	0	
		Chinese chive	132	0	
		Chive	128	0	
		Cucumber	41	0	
		Watermelon	15	0	
		Potato	3	0	
		Bean (kidney/French bean)	28	0	
		Green chili	5	0	
Costa Rica	Zielh-Nielsen and Weber stain	Lettuce	50	2 (4.0)	[71]
		Parsley	50	0	
		Cilantro	50	0	
		Strawberries	50	0	
		Blackberries	50	0	
Egypt	Weber modified trichrome and modified Ziehl-Neelsen stains	Fresh fruit juices		14.5	[80]
Ethiopia	Modifed Zeihl-Neelsen stain	Fruits and vegetables	360	18 (5.0)	[19]
Ethiopia	Modified Ziehl-Neelsen stain	Fruits and vegetables	360	25 (6.9)	[32]
Ethiopia	Modified Zeihl-Neelsen stain	Tomato	100	4 (4.0)	[14]
		Cabbage	96	0	
		Green pepper	66	2 (3.0)	
		Carrot	62	0	
		Salad	23	1 (4.5)	
Ghana	Direct wet mount, trichrome modified Ziehl-Neelsen stain	Tiger nuts	40	9 (22.5)	[81]
Ghana	Ziehl-Neelsen stain	Cabbage	90	5 (5.6)	[12]
		Green pepper	55	3 (5.5)	
		Carro	47	3 (6.4)	
		Onion	70	3 (4.3)	
		Tomato	31	3 (9.7)	
		Lettuce	102	3 (2.9)	
Italy	qPCR	Vegetables	49	6 (12.2)	[48]
Italy	modified Ziehl-Neelsen stain and PCR	Ready-to-eat packaged salad	648	8 (1.2)	[23]
Korea	Multiplex qPCR	Perilla leaves	72	0	[48]
		Winter-grown cabbage	70	4 (5.7)	
		Chives	73	0	
		Sprouts	72	1 (1.4)	
		Blueberries	44	1 (2.3)	
		Cherry tomatoes	73	1 (1.4)	
Peru	Direct microscopic observation, acid-fast staining, and immunofluorescent assay	Vegetables		1.8	[83]
Vietnam	Modified acid-fast smear by light and UV epifluorescence microscopy	Basil	96	10 (10.4)	[47]
		Coriander sativum	80	3 (3.8)	
		Coriander	86	10 (11.6)	
		Lettuce	79	8 (10.1)	
		Vietnamese mint	61	6 (9.8)	
		Marjoram	26	2 (7.7)	
		Persicaria	68	7 (10.3)	
Total			4628	180 (3.9)	

### *Entamoeba* contamination

Among the *Entamoeba* spp., *E. histolytica* is responsible for most cases of human amebiasis and remains one of the top three causes of parasitic mortality worldwide [49]. Although some of the *E. histolytica* infections are asymptomatic, many infections may lead to severe amoebic colitis and disseminated disease [50]. *Entamoeba* spp. infections are significantly associated with the consumption of contaminated vegetables and fruits [17, 41, 51, 52].

*Entamoeba* spp. cysts can be detected with light microscopy based on their morphological features [29, 42, 43]. Staining with Lugol’s iodine is widely used to detect the *Entamoeba* spp. cysts (e.g. [12, 14, 17, 19, 52]). The PCR technique is also commonly used to detect *Entamoeba* spp. in food items based on amplification and sequencing of specific genes [23, 53].

Many reports have documented the contamination of raw vegetables and fruits with *Entamoeba* spp. cysts worldwide (Table 4). The average prevalence of *Entamoeba* contamination is calculated as 3.5% (199/5647; 95% CI: 3.0–4.0%). *Entamoeba histolytica*, *E. dispar* and *E. coli* were the most commonly detected species among the isolates from contaminated vegetables and fruits [12, 17, 29, 42].

**Table 4:** Contamination of vegetables and fruits with *Entamoeba* spp.

Location	Detection method	Vegetable or fruit item	Number of samples tested	Number of positive samples (%)	*Entamoeba* species identified (*n*)	References
Bangladesh	Wet mount	Vegetables	200	17 (8.5)	*Entamoeba histolytica*	[52]
Brazil	Direct smear, followed by light microscopy	Lettuce	30	3 (10.0)	*Entamoeba coli* (3)	[85]
Brazil	Lugol’s iodine stain	Loose leaf lettuce^a^	1	1	*Entamoeba* sp.	[30]
		Red lettuce^a^	1	1	*Entamoeba* sp.	
		Curly lettuce^a^	1	1	*Entamoeba* sp.	
		Iceberg lettuce^a^	1	1	*Entamoeba* sp.	
		Parsley^a^	1	1	*Entamoeba* sp.	
		Chive^a^	1	1	*Entamoeba* sp.	
		Coriander^a^	1	1	*Entamoeba* sp.	
		Basil^a^	1	1	*Entamoeba* sp.	
		Arugula^a^	1	1	*Entamoeba* sp.	
		Chicory^a^	1	1	*Entamoeba* sp.	
		Kale^a^	1	1	*Entamoeba* sp.	
		Bean sprouts^a^	1	1	*Entamoeba* sp.	
Brazil	Sediment smear, followed by light microscopy	Vegetables	100	32 (32.0)	*Entamoeba* spp. (32)	[86]
Brazil	Sediment being stained in Lugolʼs solution	Lettuce	100	9 (9.0)	*Entamoeba histolytica* (9)	[15]
		Lettuce	100	4 (4.0)	*Entamoeba coli* (4)	
		Coriander	100	11 (11.0)	*Entamoeba histolytica* (11)	
		Coriander	100	4 (4.0)	*Entamoeba coli* (4)	
Cameroon	Lugol’s iodine stain	Green cabbage	30	5 (16.7)	*Entamoeba* spp. (5)	[66]
		Red cabbage	30	3 (10.0)	*Entamoeba* spp. (3)	
		Lettuce	30	9 (30.0)	*Entamoeba* spp. (9)	
		Cucumber	30	5 (16.7)	*Entamoeba* spp. (5)	
		Carrots	30	3 (10.0)	*Entamoeba* spp. (3)	
		Green pepper	30	5 (16.7)	*Entamoeba* spp. (5)	
Costa Rica	Direct smear, followed by light microscopy	Cilantro leaves	80	5 (6.2)	*Entamoeba histolytica* (5)	[79]
		Cilantro roots	80	2 (2.5)	*Entamoeba histolytica* (2)	
		Lettuce	80	3 (3.8)	*Entamoeba histolytica* (3)	
		Radish	80	2 (2.5)	*Entamoeba histolytica* (2)	
Egypt	Lugol’s iodine stain	Lettuce	101	14 (13.9)	*Entamoeba* spp. (14)	[18]
		Watercress	116	9 (7.8)	*Entamoeba* spp. (9)	
		Parsley	102	8 (7.8)	*Entamoeba* spp. (8)	
		Green onion	103	2 (1.9)	*Entamoeba* spp. (2)	
		Leek	108	3 (2.8)	*Entamoeba* spp. (3)	
Ethiopia	Lugol’s iodine stain	Fruits and vegetables	360	19 (5.3)	*Entamoeba histolytica*/***E.****dispar* (19)	[19]
Ethiopia	Sediment smear	Fruits and vegetables	360	52 (14.4)	*E. histolytica/dispar* (52)	[32]
Ethiopia	Lugol’s iodine stain	Tomato	100	22 (22.0)	*E. histolytica* (22)	[14]
		Cabbage	96	0		
		Green pepper	66	0		
		Carrot	62	7 (11.3)	*E. histolytica* (7)	
		Salad	23	0		
Ethiopia	Sediment smear under light microscope	Tomatoes	45	1 (2.2)	*E. histolytica/E. dispar* (1)	[43]
		Lettuce	45	4 (8.8)	*E. histolytica/E. dispar* (4)	
		Carrot	45	6 (13.3)	*E. histolytica/E. dispar* (6)	
		Cabbage	45	7 (15.6)	*E. histolytica/E. dispar* (7)	
		Green pepper	45	5 (11.1)	*E. histolytica/E. dispar* (5)	
		Avocado	45	5 (11.1)	*E. histolytica/E. dispar* (5)	
Ghana	Lugol’s iodine stain	Cabbage	90	5 (5.6)	*Entamoeba coli* (5)	[12]
		Green pepper	55	4 (7.3)	*Entamoeba coli* (4)	
		Onion	70	2 (2.9)	*Entamoeba coli* (2)	
		Tomato	31	2 (6.5)	*Entamoeba coli* (2)	
		Lettuce	102	4 (3.9)	*Entamoeba coli* (4)	
Ghana	Lugol’s iodine stain	Cabbage	90	11 (12.2)	*Entamoeba histolytica* (11)	
		Carrot	47	4 (8.5)	*Entamoeba histolytica* (4)	
		Onion	70	2 (2.9)	*Entamoeba histolytica* (2)	
		Tomato	31	4 (12.9)	*Entamoeba histolytica* (4)	
		Lettuce	102	6 (5.9)	*Entamoeba histolytica* (6)	
Iran	Lugol’s iodine stain	Vegetables	141	18 (12.8)	*Entamoeba coli* (18)	[84]
Iran	Sediment smear under light microscopy	Leek	30	0		[42]
		Spring onion	22	2 (9.1)	*Entamoeba coli* (2)	
		Basil	15	0		
		Parsley	21	0		
		Lettuce	23	0		
		Cress	17	1 (5.9)	*Entamoeba coli* (1)	
		Spearmint	18	0		
		Tarragon	19	1 (5.3)	*Entamoeba coli* (1)	
		Coriander	24	2 (8.3)	*Entamoeba coli* (2)	
		Radish	29	0		
Iran	Sediment smear under light microscopy	Leek	30	2 (6.7)	*Entamoeba histolytica* (2)	[42]
		Spring onion	22	0		
		Basil	15	0		
		Parsley	21	0		
		Lettuce	23	0		
		Cress	17	0		
		Spearmint	18	1 (5.6)	*Entamoeba histolytica* (1)	
		Tarragon	19	0		
		Coriander	24	0		
		Radish	29	0		
Iran	Lugol’s iodine stain	Vegetables	34	1 (2.9)	*Entamoeba coli* (1)	[72]
Jordan	Lugol’s iodine stain	Lettuce	30	3 (10.0)	*Entamoeba histolytica* (3)	[20]
		Tomato	33	2 (6.1)	*Entamoeba histolytica* (2)	
		Parsley	42	0		
		Cucumber	28	0		
Saudi Arabia	Lugol’s iodine stain	Green onion	50	6 (12.0)	*Entamoeba* spp. (6)	[17]
		Watercress	50	8 (16.0)	*Entamoeba* spp. (8)	
		Lettuce	50	6 (12.0)	*Entamoeba* spp. (6)	
		Cucumber	50	7 (14.0)	*Entamoeba* spp. (7)	
		Cabbage	50	6 (12.0)	*Entamoeba* spp. (6)	
		Pea	50	5 (10.0)	*Entamoeba* spp. (5)	
		Tomato	50	0		
		Carrot	50	6 (12.0)	*Entamoeba* spp. (6)	
Saudi Arabia	Lugol’s iodine stain	Green onion	50	3 (6.0)	*Entamoeba coli* (3)	
		Watercress	50	4 (8.0)	*Entamoeba coli* (4)	
		Lettuce	50	2 (4.0)	*Entamoeba coli* (2)	
		Cucumber	50	2 (4.0)	*Entamoeba coli* (2)	
		Cabbage	50	4 (8.0)	*Entamoeba coli* (4)	
		Pea	50	3 (6.0)	*Entamoeba coli* (3)	
		Tomato	50	2 (4.0)	*Entamoeba coli* (2)	
		Carrot	50	3 (6.0)	*Entamoeba coli* (3)	
Sudan	Lugol’s iodine stain	Tomatoes	36	1 (2.8)	*Entamoeba coli* (1)	[29]
		Cucumber	12	0		
		Armenian cucumber	16	0		
		Green pepper	25	0		
		Cayenne pepper	7	0		
		Radish	24	1 (4.2)	*Entamoeba coli* (1)	
		Beet	19	1 (5.3)	*Entamoeba coli* (1)	
		Watercress	23	1 (4.3)	*Entamoeba coli* (1)	
		Lettuce	11	1 (9.1)	*Entamoeba coli* (1)	
		Green onion	36	0		
		Carrot	50	0		
Sudan	Lugol’s iodine stain	Tomatoes	36	1 (2.8)	*Entamoeba* spp. (1)	[29]
		Cucumber	12	0		
		Armenian cucumber	16	2 (12.5)	*Entamoeba* spp. (2)	
		Green pepper	25	1 (4.0)	*Entamoeba* spp. (1)	
		Cayenne pepper	7	0		
		Radish	24	0		
		Beet	19	1 (5.3)	*Entamoeba* spp. (1)	
		Watercress	23	1 (4.3)	*Entamoeba* spp. (1)	
		Lettuce	11	2 (18.2)	*Entamoeba* spp. (2)	
		Green onion	36	4 (11.1)	*Entamoeba* spp. (4)	
		Carrot	50	3 (6.0)	*Entamoeba* spp. (3)	
Total			5647	199 (3.5)		

^a^Single sample in a case report

### *Toxoplasma gondii* contamination

*Toxoplasma gondii* is a ubiquitous protozoan parasite capable of infecting virtually all warm-blooded animals [54]. According to a new nomenclature system, *T. gondii* genotypes are classified as Type I, Type II or Type III. Other atypical or exotic genotypes include Chinese 1, Type Br I, Type Br II, Type Br III, Type IV and Type 12 [55, 56]. Among the three principal routes of toxoplasmosis transmission, consumption of unwashed vegetables and fruits contaminated with cat feces is an important one that sometimes may lead to food-borne outbreaks [57]. The significant association of *T. gondii* infections with the consumption of contaminated raw vegetables is also observed in previous studies [58–60].

The detection of *Toxoplasma gondii* in contaminated vegetables and fruits is usually performed by PCR amplification [23, 61–63]. The contamination of vegetables and fruits with *T. gondii* was observed in Brazil, China, Italy and Poland (Table 5), and the average prevalence of the contamination was estimated as 3.8% (63/1676; 95% CI: 2.9–4.7%). The *T. gondii* isolates obtained from vegetables and fruits belonged to genotypes Type I and II [23, 61, 64].

**Table 5:** Contamination of vegetables and fruits with *Toxoplasma gondii*

Location	Detection method	Vegetable or fruit item	No. of samples tested	No. of positive samples (%)	*Toxoplasma gondii* genotypes identified (*n*)	References
Brazil	PCR	Smooth lettuce	62	1 (0.6)	Toxo4-5 D (1)	[62]
		Crisp head lettuce	106	4 (3.7)	B22-23 D (4)	
		Chicory	40	2 (5.0)	B22-23 D (1); Toxo4-5 D (1)	
		Rocket	7	1 (14.3)	B22-23 D (1)	
		Parsley	5	1 (20.0)	B22-23 D (1)	
Brazil	PCR	Vegetables	21	3 (14.3)	N/A (3)	[45]
China	Quantitative real-time PCR (qPCR)	Lettuce	71	5 (7.0)	Type I (4); Type II (1)	[63]
		Spinach	50	2 (4.0)	Type I (2)	
		Pak choi	34	1 (2.9)	Type I (1)	
		Chinese cabbage	26	0		
		Rape	22	1 (4.5)	Type II (1)	
		Asparagus	18	0		
		*Chrysanthemum coronarium*	16	0		
		Endive	14	0		
		Chinese chives	11	0		
		Cabbage	9	0		
		Red cabbage	8	1 (12.5)	Type II (1)	
Czech Republic	Triplex real time PCR	Carrots	93	7 (7.5)		[64]
		Cucumbers	109	13 (11.9)	Type II (5)	
		Salads	90	8 (8.9)	Type II (2)	
Italy	qPCR	Ready-to-eat packaged salad	648	5 (0.8)	Type I (5)	[23]
Poland	qPCR	Strawberries	60	0		[61]
		Radish	60	3 (5.0)	Type I (2); Type II (1)	
		Carrot	46	9 (19.6)	Type I (3); Type II (1)	
		Lettuce	50	9 (18.0)	Type I (1)	
Total			1676	63 (3.8)		

### Other intestinal protozoan contaminations

Fresh vegetables and fruits are occasionally contaminated with some other intestinal protozoans, such as *Balantioides coli*, *Cystoisospora belli*, *Blastocystis* sp. and *Enterocytozoon bieneusi*.

Several reports have documented *B. coli* contamination of vegetables, leading to global public health concerns [65]. *Balantioides coli* is usually detected on vegetables and fruits with light microscopy [14, 30, 52, 66, 67]. The contamination of vegetables with *B. coli* has been reported in Bangladesh, Brazil, Cameroon, Ethiopia, and Ghana (Table 6) and the average prevalence of the contamination is calculated as 9.3% (72/907; 95% CI: 7.6–11.0%).

**Table 6:** Contamination of vegetables and fruits with *Balantidium coli*, *Cystoisospora belli*, *Blastocystis* sp. and *Enterocytozoon bieneusi*

Location	Detection method	Vegetable or fruit item	No. of samples tested	No. of positive samples (%)	Identified species or genotypes (*n)*	References
*Balantidium coli*						
Bangladesh	Sediment smears, followed by light microscopy	Vegetables	200	8 (4.0)	*B. coli*	[52]
Brazil	Sediment smears, followed by light microscopy	Loose leaf lettuce^a^	1	1	*B. coli*	[30]
		Red lettuce^a^	1	1	*B. coli*	
		Curly lettuce^a^	1	1	*B. coli*	
		Iceberg lettuce^a^	1	1	*B. coli*	
		Parsley^a^	1	1	*B. coli*	
		Chive^a^	1	1	*B. coli*	
		Coriander^a^	1			
Cameroon	Sediment smears, followed by light microscopy	Green cabbage	30	3 (10.0)	*B. coli* (3)	[66]
		Red cabbage	30	7 (23.3)	*B. coli* (7)	
		Lettuce	30	8 (26.7)	*B. coli* (8)	
		Cucumber	30	5 (16.7)	*B. coli* (5)	
		Carrots	30	4 (13.3)	*B. coli* (4)	
		Green pepper	30	2 (6.7)	*B. coli* (2)	
Ethiopia	Sediment smears, followed by light microscopy	Tomato	100	0		[14]
		Cabbage	96	4 (4.2)	*B. coli-*like (4)	
		Green pepper	66	6 (9.1)	*B. coli-*like (6)	
		Carrot	62	4 (6.5)	*B. coli-*like (4)	
		Salad	23	1 (4.3)	*B. coli-*like (1)	
Ghana	Sediment smears, followed by light microscopy	Cabbage	72	21 (29.2)	*B. coli* (21)	[67]
		Lettuce	72	3 (4.2)	*B. coli* (3)	
		Carrot	72	2 (2.8)	*B. coli* (2)	
		Spring onion	72	1 (1.4)	*B. coli* (1)	
		Tomatoes	72	22 (30.6)	*B. coli* (22)	
Subtotal			1087	101 (9.3)		
*Cystoisospora belli*						
Ethiopia	Modified Ziehl-Neelsen stain	Fruits and vegetables	360	11 (3.1)	*I. belli* (11)	[32]
Ethiopia	Modified Ziehl-Neelsen stain	Tomatoes	45	0		[43]
		Lettuce	45	1 (2.2)	*C. belli* (1)	
		Carrot	45	2 (4.4)	*C. belli* (2)	
		Cabbage	45	4 (8.8)	*C. belli* (4)	
		Green pepper	45	0		
		Avocado	45	0		
Ghana	Ziehl-Neelsen stain	Cabbage	90	0		[12]
		Green pepper	55	0		
		Carro	47	0		
		Onion	70	0		
		Tomato	31	1 (3.2)	*I. beli* (1)	
		Lettuce	102	0		
Subtotal			1025	19 (1.9)		
*Blastocystis* sp.						
Brazil	Sediment being stained in Lugolʼs solution	Lettuce	100	15 (15.0)	*B. hominis* (15)	[15]
		Coriander	100	19 (19.0)	*B. hominis* (19)	
Italy	Lugolʼs stain, Giemsa Stain, and PCR	Ready-to-eat packaged salad	648	3 (0.5)	*B. hominis* (3)	[23]
Subtotal			848	37 (4.4)		
*Enterocytozoon bieneusi*						
China	PCR	Lettuce	200	14 (7.0)	*E. bieneusi* genotype CM8 (2); CD6 (7); EbpA (3); Henan-IV (1)	[36]
		Coriander	152	1 (0.7)	*E. bieneusi* genotype CM8 (1)	
		Celery	70	1 (1.4)	*E. bieneusi* genotype EbpA (1)	
		Baby bok choy	59	1 (1.7)	*E. bieneusi* genotype CHV3 (1)	
		Chinese cabbage	47	0		
		Leaf lettuce	44	2 (4.5)	*E. bieneusi* genotype CHG19 (1)	
		Water spinach	28	3 (10.7)	*E. bieneusi* genotype CD6 (1); BEB8 (1); CTS3 (1)	
		Crown daisy	27	0		
		Fennel plant	26	1 (3.9)	*E. bieneusi* genotype EbpC (1)	
		Endive	25	1 (4.0)	*E. bieneusi* genotype Henan-IV (1)	
		Spinach	20	0		
		Schizonepeta	20	0		
		Cabbage	18	0		
		Leaf mustard	11	0		
		Chinese chive	132	6 (4.5)	*E. bieneusi* genotype CD6 (1); EbpA (2); EbpC (1); CHV1 (1)	
		Chive	128	4 (1.4)	*E. bieneusi* genotype CD6 (2); CHV2 (1); CTS3 (1)	
		Cucumber	41	1 (2.4)	*E. bieneusi* genotype CD6 (1)	
		Watermelon	15	1 (6.7)	*E. bieneusi* genotype CD6 (1)	
		Potato	3	1 (33.3)	*E. bieneusi* genotype CHV4 (1)	
		Bean (kidney/French bean)	28	4 (14.3)	*E. bieneusi* genotype CD6 (4)	
		Green chili	5	0		
Costa Rica	Zielh-Nielsen stain	Lettuce	50	16 (32.0)	*E. bieneusi* (16)	[71]
		Parsley	50	0		
		Cilantro	50	2 (4.0)	*E. bieneusi* (2)	
		Strawberries	50	1 (2.0)	*E. bieneusi* (1)	
		Blackberries	50	0		
Poland	Conventional stain and FISH	Berries	25	6 (24.0)	*E. intestinalis* (4); *E. bieneusi* (2)	[21]
		Sprouts	20	1 (5.0)	*E. bieneusi* (1)	
		Vegetables	35	2 (5.7)	*E. cuniculi* (1); *E. bieneusi* (1)	
Sub-total			1429	52 (3.6)		

^a^Single sample in a case report

*Cystoisospora belli* infection is commonly reported in tropical and subtropical areas of the world [68]. Cystoisosporiasis can be acquired through the ingestion of contaminated food. *Cystoisospora belli* is commonly detected with modified Ziehl-Neelsen staining, followed by microcopy [32, 43]. There are three reports on *Cystoisospora belli* contamination in vegetables and fruits in Ethiopia and Ghana (Table 6). The average prevalence of the contamination is estimated as 1.9% (19/1025; 95% CI: 1.1–2.7%).

The detection of *Blastocystis* sp. is usually based on microscopy and PCR [23]. Cell culture is also used for the detection of this parasite. The contamination of vegetables and fruits with *Blastocystis* sp. has only been documented in Brazil and Italy, with a prevalence of 4.4% (37/848; 95% CI: 3.0–5.8%) (Table 6).

*Enterocytozoon bieneusi* is an important microsporidian species infecting humans [69]. The genetic diversity of the pathogen is inferred by the analysis of single nucleotide polymorphisms (SNPs) in the internal transcribed spacer (ITS) that resulted in nearly 500 valid genotypes of the pathogen [70]. The phylogenetic analysis of the valid genotypes recognized eleven genetic groups (Groups 1 to 11), figuring out their host specificity and zoonotic potential. Food-borne transmission of *E. bieneusi* has been documented and the contamination of vegetables and fruits with this pathogen was reported in China, Costa Rica and Poland (Table 6). The parasite was successfully detected in contaminated vegetables and fruits by staining or with fluorescence *in situ* hybridization [21, 71], and PCR amplification [36]. The average prevalence of the reported contamination was estimated as 3.6% (52/1429; 95% CI: 2.6–4.6%).

### Risk factors involved in the contamination of vegetables and fruits with parasites

Previous studies in Ethiopia, Ghana, Brazil and Iran reported a relatively higher prevalence of intestinal parasitic infections associated with the consumption of vegetables sold at open-aired markets than those associated with supermarkets [12, 14, 15]. The parasitic load in the raw vegetables of open markets was high and posed a high risk of parasitic infections. The high contamination rates recorded in the open-market samples indicate poor hygiene in these locations, which is suitable for the propagation and transmission of the parasites [72].

High risk of diarrhea among raw vegetable consumers in the Kathmandu valley of Nepal, mostly due to the use of river water by farmers for washing vegetables, suggests a need to avoid the use of river water for washing vegetables [73]. There are also many reports that highlight the contamination of surface water with parasitic infective stages in Brazil [74], Iran [75], Poland [76] and Spain [77]. The use of such contaminated surface water for washing fresh vegetables and fruits might cause parasitic contamination.

Another study in the Czech Republic reported a significantly higher contamination of *T. gondii* in vegetables collected from farm storage rooms than those from fields [64], indicating a higher chance of contamination of vegetables and fruits during processing and selling [78]. Therefore, the adaptation of good practices in every step between farm and fork, such as production, processing, storage and selling minimize the microbial contamination of vegetables and fruits.

## Conclusions

The accidental ingestion of parasitic infective stages such as eggs, oocysts, cysts or spores with the contaminated raw vegetables or fruits causes varying intestinal diseases in humans that sometimes may lead to serious health problems. On many occasions, the contamination of vegetables and fruits results in outbreaks of the parasitic diseases. Globally, the occurrence of protozoan parasitic contamination in vegetables and fruits ranges from 1.9% to 9.3%. However, contamination with protozoans may be grossly underestimated, especially in regions with poor sanitation. Contamination of vegetables and fruits with parasites can occur in many ways. The common stages between farm and fork at which vegetables and fruits are contaminated include production, processing, storage and selling. Therefore, the implementation of hygienic practices at every step between production and consumption may eliminate the contamination. The appropriate local public health authority is recommended to establish a system for continuous monitoring of contamination of vegetables and fruits sold at local markets.

## Data Availability

All data generated or analysed during this study are included in this published article.

## Abbreviations

CI: confidence interval
ITS: internal transcribed spacer
PCR: polymerase chain reaction
SNP: single-nucleotide polymorphism
